# Draft Genome Sequence of Biocontrol Bacterium *Brevibacillus brevis* Strain FJAT-0809-GLX

**DOI:** 10.1128/genomeA.00160-13

**Published:** 2013-04-25

**Authors:** Jianmei Che, Bo Liu, Yingzhi Lin, Weiqi Tang, Jianyang Tang

**Affiliations:** Agricultural Bio-resource Institute, Fujian Academy of Agricultural Sciences, Fuzhou, Fujian, China

## Abstract

*Brevibacillus brevis* strain FJAT-0809-GLX had significant inhibition on many plant and animal pathogens. The draft genome sequence of *B. brevis* FJAT-0809-GLX is 6 Mb in size and consists of 5,677 genes (protein-coding sequences [CDS]), with an average length of 933 bp and a G+C content of 47.30%. Compared with the published *B. brevis* strain NBRC 100599, 618 specific genes were identified in the strain FJAT-0809-GLX.

## GENOME ANNOUNCEMENT

*Brevibacillus brevis* (formerly *Bacillus brevis* Nagano [1]) is a Gram-positive and spore-forming bacterium (2). It can secrete large amounts of secondary metabolites, which are important for controlling pathogens. Among them, tyrocidine (3), gramicidin (4–6), gratisin (7), and edeine (8) have been successively isolated and identified. *B. brevis* could be a promising candidate for use as an antimicrobial agent to improve crop yield and quality, owing to its antagonistic activities against pathogenic bacteria and fungi (2). The first genome of *B. brevis*, strain NBRC 100599, is available at GenBank. Here, we report the second genome for *B. brevis*, strain FJAT-0809-GLX.

*B. brevis* FJAT-0809-GLX was isolated from the soil of watermelon rhizosphere in Fujian province, China. It was identified by 16S rRNA gene sequencing and physiological and biochemical analyses (9). Studies showed that it had significant inhibition on many plant and animal pathogens (10–13), such as *Ralstonia solanacearum* (10), *Fusarium oxysporum* (10,11), and *Escherichia coli* K88 (13). It also had an inhibitory effect on the activity of peroxidase (POD) from longan pericarp for keeping fruit fresh (13). Here, we analyzed the genome sequence of this strain to explore the genomic features responsible for its effectiveness as a biocontrol agent.

The genome of *B. brevis* FJAT-0809-GLX was sequenced by Illumina GA IIx with a 400-bp paired-end library. Using SOAP*denovo* v1.04 (14), a total of 739 Mb high-quality bases were *de novo* assembled into 72 contigs, with a total genome size of 6.02 Mb, of which the N_50_ is 181 kb. The G+C content of the FJAT-0809-GLX genome is approximately 47.3%.

In all, 5,677 coding sequences (CDSs) were predicted by Prodigal v2.5 (15), with a mean gene length of 933 bp. The CDSs represent 87.8% of the genome. Functional annotation of the predicted genes was performed by homologous comparison to NR and UniRef (BLASTp, *e* value ≤ 1E - 5); however, 362 genes did not match any known protein in a current public database. Of the total proteins, 3,830 can be assigned to COG families and 2,048 can be assigned to KEGG. Additionally, 1 rRNA and 80 tRNAs were identified by RNAmmer (16) and tRNAscan-SE 1.21 (17), respectively.

Compared with the sequenced genome of *B. brevis* strain NBRC 100599, 618 strain-specific genes were identified within the genome of strain FJAT-GLX-0809, accounting for 10.89% of the total number of predicted genes, most of which encoded hypothetical and putative uncharacterized proteins. The average amino acid identity (AAI) of orthologous genes is 97.1% between the two strains. The *B. brevis* FJAT-0809-GLX genome might not only enrich the genome database of *B. brevis*, but it might also lay the foundation at the genomic level for the research of antimicrobial mechanisms and the development of related products.

### Nucleotide sequence accession numbers. 

The whole-genome shotgun project for *B. brevis* FJAT-0809-GLX has been deposited at DDBJ/EMBL/GenBank under the accession no. AHKL00000000. The version described in this paper is the first version, accession no. AHKL01000000.
